# Quantitative Proteomics Reveals Cellular Targets of Celastrol

**DOI:** 10.1371/journal.pone.0026634

**Published:** 2011-10-26

**Authors:** Jakob Hansen, Johan Palmfeldt, Søren Vang, Thomas J. Corydon, Niels Gregersen, Peter Bross

**Affiliations:** 1 Research Unit for Molecular Medicine, Aarhus University Hospital, Skejby, Aarhus, Denmark; 2 Department of Forensic Medicine, Aarhus University, Aarhus, Denmark; 3 Department of Molecular Medicine, Aarhus University Hospital, Skejby, Aarhus, Denmark; 4 Department of Human Genetics, Aarhus University, Aarhus, Denmark; Ludwig-Maximilians University, Germany

## Abstract

Celastrol, a natural substance isolated from plant extracts used in traditional Chinese medicine, has been extensively investigated as a possible drug for treatment of cancer, autoimmune diseases, and protein misfolding disorders. Although studies focusing on celastrol's effects in specific cellular pathways have revealed a considerable number of targets in a diverse array of *in vitro* models there is an essential need for investigations that can provide a global view of its effects. To assess cellular effects of celastrol and to identify target proteins as biomarkers for monitoring treatment regimes, we performed large-scale quantitative proteomics in cultured human lymphoblastoid cells, a cell type that can be readily prepared from human blood samples. Celastrol substantially modified the proteome composition and 158 of the close to 1800 proteins with robust quantitation showed at least a 1.5 fold change in protein levels. Up-regulated proteins play key roles in cytoprotection with a prominent group involved in quality control and processing of proteins traversing the endoplasmic reticulum. Increased levels of proteins essential for the cellular protection against oxidative stress including heme oxygenase 1, several peroxiredoxins and thioredoxins as well as proteins involved in the control of iron homeostasis were also observed. Specific analysis of the mitochondrial proteome strongly indicated that the mitochondrial association of certain antioxidant defense and apoptosis-regulating proteins increased in cells exposed to celastrol. Analysis of selected mRNA transcripts showed that celastrol activated several different stress response pathways and dose response studies furthermore showed that continuous exposure to sub-micromolar concentrations of celastrol is associated with reduced cellular viability and proliferation. The extensive catalog of regulated proteins presented here identifies numerous cellular effects of celastrol and constitutes a valuable biomarker tool for the development and monitoration of disease treatment strategies.

## Introduction

Plants used in traditional Chinese medicine are rich sources of biologically active substances with potential therapeutic effects towards many human diseases [1]. Root bark extracts of the plant thunder of god vine (*Tripterygium wilfordii*) have been used in traditional Chinese medicine to treat fever, inflammation, and various other health problems in man [2]. Celastrol is a key constituent of thunder of god vine and possesses anti-inflammatory activities that have shown promising therapeutic effects in models of Alzheimer's disease [3], amyotrophic lateral sclerosis [4], and rheumatoid arthritis [5], [6]. Numerous studies have furthermore shown that celastrol inhibits proliferation of various cancer cell lines *in vitro* and suppresses tumor growth in animal cancer models, as reviewed in [7], [8]. Studies in a cellular model of the inherited lysosomal storage disorder Gaucher's disease furthermore suggest that celastrol may be used to ameliorate diseases caused by protein misfolding through enhancing cellular protein folding and trafficking [9]. The numerous indications of therapeutic effects in model systems have stimulated investigations into the underlying molecular mechanisms. Celastrol modulates the expression of genes regulated through the (NF)-κΒ system [10], [11]. This system regulates multiple cellular activities related to the immune system and its deregulation is linked to inflammatory diseases and cancer [12]. Celastrol also triggers the heat shock response [13], a stress response pathway that induces the expression of heat shock proteins to protect cells from damage associated with heat-induced protein misfolding [14]. Modulation of stress response pathways and the resulting increased expression of molecular chaperones in different cellular compartments may explain why celastrol protects cells against an otherwise lethal severe heat shock exposure [13] and improves intracellular folding and processing of mutated proteins traversing the endoplasmic reticulum [9]. It may seem controversial that a single agent like celastrol can have therapeutic potential towards several human diseases of different etiologies and it certainly warrants further investigations into its molecular targets and cellular effects. To provide a global view of the multiple effects of celastrol we here report an extensive analysis of celastrol-regulated proteins in cultured human lymphoblastoid cells based on large-scale quantitative proteomics using mass spectrometry and stable isotope labeling with amino acids in cell culture (SILAC) [15]. This analysis revealed a large number of celastrol-regulated proteins, including many previously unrecognized targets, and it significantly expands the current view of the multifaceted effects of celastrol.

## Results and Discussion

### Overview of SILAC approach and quantified proteins

**Figure 1:**
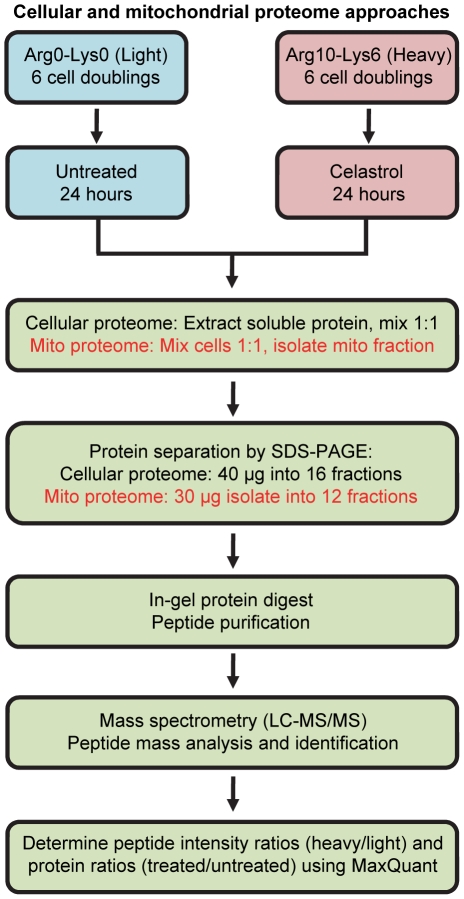
Schematic overview of the SILAC-based experimental design.

Stable isotope labeling with amino acids in cell culture (SILAC) was used to differentially label the proteomes of two cell populations, which subsequently were incubated in either 0.8 µM celastrol (treated) or in vehicle alone (dimethyl sulfoxide; untreated) for 24 h (Figure 1). This dose was chosen because it effectively increased the mRNA levels of *HSPA1B* (a well-known celastrol targets encoding the cytosolic heat shock protein Hsp70) and *HSPD1* (encoding the mitochondrial heat shock protein 60 (Figure S1). Although the induction of *HSPA1B* peaked at celastrol concentrations of approximately 5 µM a lower but still effective concentration was preferred because of the well-known cytotoxic effect of this drug. Following treatment, cell populations were processed according to two different methodologies focusing on the cellular proteome or the mitochondrial proteome specifically (Figure 1). Relative changes in protein levels following celastrol treatment (the treated to untreated ratio) were calculated from nanoLC-MS/MS analysis of tryptic peptides using the MaxQuant quantification algorithms [16].

**Figure 2:**
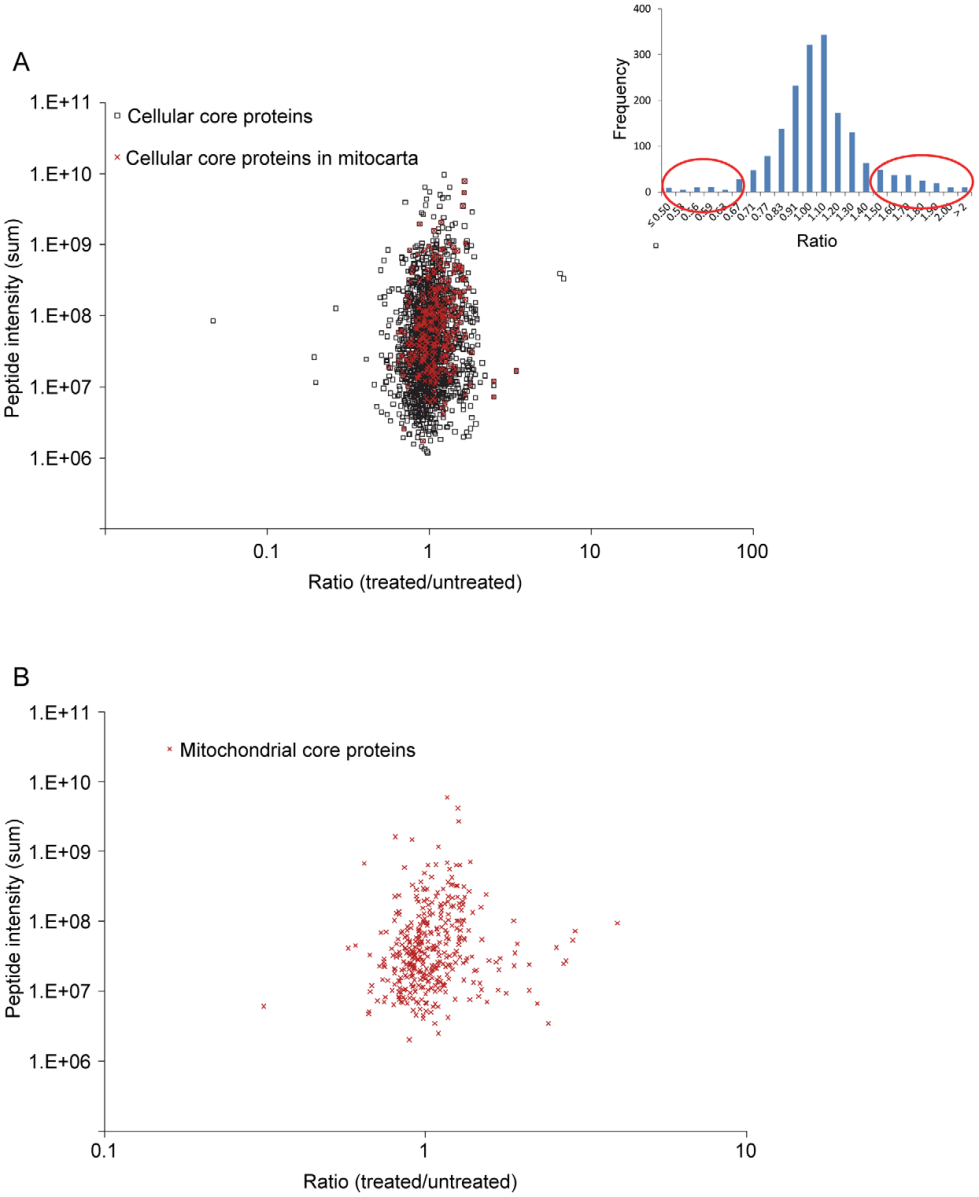
Overall distribution of quantitative protein ratios within the cellular proteome study. (A) The mean treated/untreated ratios for the 1779 cellular core proteins, including 314 of mitochondrial origin (according to human MitoCarta; marked with red cross). The figure insert shows the frequency distribution of the protein ratios (x-axis legends define interval upper limits) and proteins up- or down-regulated at least 1.5 fold are marked by oval circles. Protein ratios were calculated as the median of all SILAC peptide pair ratios identified for a given protein using normalized peptide ratios. (B) The mean treated/untreated ratios for the 375 core mitochondrial proteins. Ratios for mitochondrial core proteins were calculated using peptides intensity ratios that were normalized using only data from proteins listed in MitoCarta.

The cellular proteome was defined from three replicated experiments in which 2765 different proteins were quantified in total (Table 1, and see Table S1 for full list). The 1779 proteins common to all three experiments were designated the “cellular core proteins” (Table 1 and full list in Table S2). The coefficient of variation (CV) for the mean ratio of the cellular core proteins was below 20% for more than 90% of the proteins, and below 10% for more than 70% of the proteins (Figure S2). We found more up-regulated than down-regulated cellular core proteins (Figure 2A). One hundred and fifty-eight cellular core proteins were altered at least 1.5 fold (listed in Table S3) and 112 of these were up-regulated and 46 were down-regulated by celastrol.

**Table 1:** Overview of proteins quantified in cellular and mitochondrial proteome approaches.

	Identified Proteins*	Proteins with quantitative data¤	Core proteins	Mitocarta proteins≠	Core mitocarta proteins	Proteins changed at least 1.5-fold#
**Cellular proteome**	3342	2765	1779	482	314	158
**Mitochondrial proteome**	2404	1822	1083	498	375	33

*Excluding proteins judged as contaminants or identified in the reversed decoy database.

¤ A quantitative SILAC protein ratio (treated/untreated) was calculated from at least two valid peptide ratio counts.

≠ Based on reference to human Mitocarta, a compendium of genes encoding proteins with solid evidence of mitochondrial origin.

# Inclusion criteria: t-test for mean ratio different from 1 (p<0.05), and mean ratio ≥1.5 or mean ratio ≤0.67.

In the mitochondrial proteome analysis, 498 different quantified proteins were classified as mitochondrial by their listing in Human Mitocarta [17] (Table 1 and full list in Table S4), and 375 of these were quantified repeatedly (Figure 2B, see full lists in Tables S5 and S6). We defined these 375 proteins as the “mitochondrial core proteins” and cross reference to the cellular proteome data revealed that 253 of these were quantified in both approaches. Our data clearly demonstrate that celastrol mediates substantial changes to the proteome composition and the high number of quantified proteins enables a solid and broad analysis of affected biological pathways.

### Overview of the functional classes of proteins affected by celastrol

We assessed whether specific biological characteristics apply to the celastrol-regulated proteins by using the functional annotations of genes provided by the Gene ontology (GO) consortium [18]. GO terms are hierarchically ordered below the general categories: cellular component, biological process, and molecular function. Overall, the quantified proteins of the present study represent a wide selection of cellular functions as illustrated by identification of protein members in 24 of the 28 GO categories hosted at the level directly below the general category “biological processes”. The GO categories in which we did not identify proteins have few protein members or represent signaling and regulatory categories containing many low abundant proteins detected less efficiently in proteome analysis.

**Figure 3:**
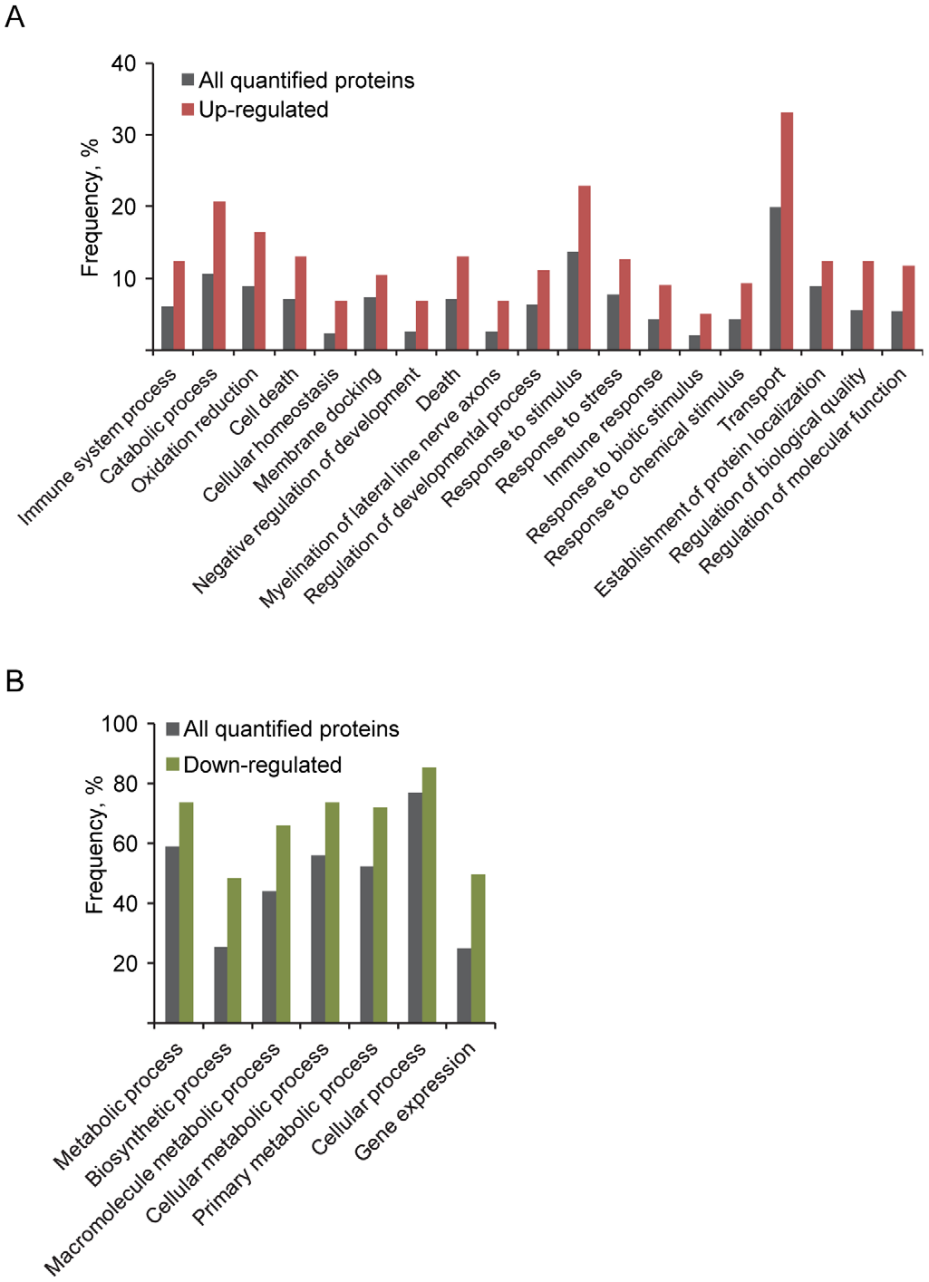
Functional GO categories in which celastrol-regulated proteins are relatively overrepresented. (A) The distribution frequencies for up-regulated proteins (red bars) into GO categories compared to the corresponding frequencies of the entire collection of proteins quantified in the cellular proteome study (gray bars). Only Go categories in which a Pearson Chi-Square statistical test indicates a significant overrepresentation of the up-regulated proteins are displayed. (B) Distribution of down-regulated proteins (green bars) into GO categories, analyzed as described in (A).

To assign functional properties specifically to the celastrol-regulated proteins (cellular core proteins with ratio different from 1 by t-test), we performed a focused comparison of their GO annotation with the annotation of all quantified proteins. Proteins up-regulated by celastrol were overrepresented in 19 different “biological process” GO categories (Figure 3A), indicating that many different biological pathways are affected by this drug. We found overrepresentation of up-regulated proteins in GO categories related to biotic and chemical stimuli. The GO analysis indicated that celastrol-treated cells shift to a more catabolic state characterized by up-regulation of proteins involved in catabolic processes (Figure 3A) and down-regulation of proteins involved in cellular metabolism, biosynthesis, and gene expression processes (Figure 3B). Finally, up-regulated proteins are also typically involved in cellular homeostatic processes, response to stress, cell death, as well as in intracellular transport processes (Figure 3A).

To analyze effects at the level of single proteins we manually grouped the quantified proteins into functional classes (Tables 2 and 3) based on knowledge of their biological functions extracted from the literature, the Uniprot protein knowledge database [19], and the GO database. To increase the confidence level we only focused on core proteins altered at least 1.5-fold. One hundred and nine of these celastrol-regulated proteins clustered into the functional pathways listed in Tables 2 and 3. All the 158 regulated cellular core proteins and the 34 mitochondrial core proteins are listed in Tables S3 and S6.

**Table 2:** Functional categorization of up-regulated proteins.

Functional pathway	Gene name	Ratio (treated/untreated) [Group range]/median
Mediators of ER protein homeostasis, ER protein quality control, and trafficking	*ERAP1*, *UGCGL1*, *GANAB*, *LMAN1*, *PRKCSH*, *LMAN2*, *SURF4*, *STT3A*, *HSP90B1*, *PDIA3*, *P4HB*, *PDIA6*, *PDIA4*, *DERL1*, *CANX*, *CALR*, *ERO1L*, *RPN1*, *DDOST*, *HSPA5*, *ERP29*, *SEC11C*, *GCS1*	[1.5 ; 2.0]/1.7
Protein quality control in other cell compartments	*HSPA1B*, *UCHL1*, *HSPB1*, *POMP*, *HSPD1*	[1.6 ; 6.7]/1.9
Antioxidant defense	*HMOX1*, ***PRDX1***, ***TXN***, *TXNDC4*, *SELT*, *HYOU1*, *SEP15*, *PRDX4*, *IFI30*	[1.5 ; 25.0]/1.9
Iron metabolism	*FTH1*, *PIR*, *PGRMC2*, *SFXN1*, *TFRC*, *BLVRB*	[1.5 ; 2.5]/1.6
Mitochondrial energy production and metabolism	***MTHFD1***, ***GARS***, *MT-ATP6*, ***LDHB***, *CYB5B*, *NNT*, *MT-CO2*, *COX4I1*, *CYB5B*, *ATP5B*, *ATP5A1*, *ATP5L*, *ATP5H*, *COX4I1*, *UQCRC1*, *ATP5C1*, *UQCRC2*, *UQCRQ*, *ATP1A1*	[1.5 ; 2.9]/1.6
Cell death and proliferation	*CTSD*, ***RHOA***, *PDCD4*, *PACAP*, *MS4A1*, *RTN3*, *VDAC1*, *LY75*, ***BAX***, ***BCL2***, *TIRAP3*, *PDCD6*, ***HK1***	[1.5 ; 2.5]/1.8

Footnote: Proteins included fulfill the following criteria: Quantified in all three replicates with a mean ratio different from 1 (t-test; p<0.05), and up- or down-regulated at least 1.5 fold by celastrol. Proteins are identified by their corresponding gene name. Proteins quantified in the mitochondrial proteome approach are marked by bold font. Median ratio and range are given for groups of functionally-related proteins.

**Table 3:** Functional categorization of down-regulated proteins.

Functional pathway	Gene name	Ratio (treated/untreated) [Group range]/median
Cell division	*CENPM*, ***TBRG4***, *UBE2C*, *RCC2*, *KIF14*	[0.2 ; 0.6]/0.5
DNA replication, transcription, repair, and translation	*RRM1*, ***TFB2M***, *MSH2*, *ASF1B*, *EIF1*, ***SHMT2***, *TSR1*, *PARP14*, ***GRSF1***, *POLD1*, *PRKDC*, *INTS3*, *RRM2*, *FEN1*, *PARP1*, *EIF5B*	[0.5 ; 0.7]/0.6
Protein sorting	*EHD4*, *AP1G2*, *ABCF2*, *KPNA2*, *SRPR*	[0.5 ; 0.7]/0.6
RNA transport and structure	*XPO5*, *DDX3X*, *DDX21*, *SERBP1*	[0.5 ; 0.6]/0.6
Proteins containing Iron/sulfur groups	*HN1*, ***ISCA2***, *RRM2*, *HBA1*	[0.3 ; 0.7]/0.5

See footnote in Table 2.

Celastrol up-regulated the cytoplasmic Hsp70 (*HSPA1B*) and Hsp27 (*HSPB1*) heat shock proteins, which are well-documented targets induced through activation of the heat shock response [13], [20]. We identified Hsp60 as a novel mitochondria-localized heat shock protein up-regulated by celastrol (Table 2). The promoter region of the *HSPD1* gene encoding Hsp60 contains regulatory regions for binding of transcription factors controlling both the heat shock response [21] and the mitochondrial unfolded protein response [22]. However, Hsp60 was most likely up-regulated through heat shock response activation since we did not observe increased ClpP protein level, which is a hallmark of mitochondrial unfolded protein response activation [23].

A long list of up-regulated proteins involved in ER protein quality control and handling activities such as folding, maturation, and sorting of proteins destined for secretion was identified (Table 2). These included the ER molecular chaperones GRP78 (*HSPA5*), Grp94 (*HSP90B1*), calnexin (*CANX*), calreticulin (*CALR*), ERp29 (*ERP29*), multiple protein disulfide-isomerases, as well as several glucosidase and glycosyltransferase enzymes involved in adding and trimming of sugar residues on ER-traversing glycoproteins. The cellular expression of several of these ER proteins is under control of the ER unfolded protein response, a well-described stress response activated by accumulation of misfolded proteins and various other cell stressors. The ER unfolded protein response signaling cascade initiates multiple events to restore ER protein homeostasis and function, including attenuation of protein translation to reduce the load of newly synthesized proteins on the ER and transcriptional activation of genes involved in restoring normal ER function, such as molecular chaperones [24]. Celastrol-mediated activation of the ER unfolded protein response may thus enhance the protein handling capacity of the ER. In relation to this, celastrol has been shown to enhance the ER trafficking and partially rescue the activity and function of mutated variants of the glucocerebrosidase enzyme that normally misfolds and are prematurely degraded [9]. This study furthermore documented that celastrol activated the ER unfolded protein response in human fibroblast cells and increased expression of the ER chaperone protein GRP78 but not of the ER chaperones GRP94 and calreticulin [9], which we identified as celastrol-regulated proteins in this study. Despite this discrepancy, both studies support the conceptual idea that the stress response-modifying activity of celastrol may be exploited to ameliorate diseases caused by misfolding of mutant proteins passing through the secretory pathway.

Proteins involved in the cellular defense against oxidative stress represented another well-defined group of celastrol-induced proteins (Table 2) that included peroxiredoxins, thioredoxins, and heme oxygenase 1 (HO-1). Peroxiredoxins catalyze the reduction of peroxides and inhibit their potential damaging reaction with other cellular constituents [25], whereas thioredoxins participate in dithiol-disulfide exchange reactions and manage redox-controlled activities [26]. Of special interest, peroxiredoxin IV has recently been shown to localize to the ER where it metabolizes hydrogen peroxide produced by the ER oxidase Ero1 during oxidative folding and thus protects cells against oxidative damage [27]. It has been suggested that peroxiredoxin proteins also function as molecular chaperones whose affinity for unfolded target proteins is directly controlled by the oxidation status of its redox-sensitive cysteines [28].

The HO-1 (*HMOX1*) was the single most (25 fold) highly up-regulated protein in our study and this protein has recently also been identified as a celastrol target in vascular smooth muscle cells [29]. The HO-1 enzyme is responsible for the intracellular oxidation of heme groups into free iron, carbon monoxide, and biliverdin [30]. The expression of heme oxygenase 1 is induced by several different stress stimuli such as increased levels of heme and oxidants like hydrogen peroxide as well as by thiol-reactive substances [31]. Increased heme oxygenase activity and the associated production of free iron is functionally linked to another group of up-regulated proteins identified in our study, namely those involved in iron binding and storage (e.g., ferritin and pirin) as well as in iron transport (e.g., sideroflexin-1 and transferring receptor protein, see Table 2). These proteins collectively participate in securing iron homeostasis and thus inhibit free iron catalyzed production of highly damaging and reactive oxygen species (ROS).

Another group of up-regulated proteins is collectively involved in mitochondrial energy production and oxidative phosphorylation process (Table 2). Of special interest, several up-regulated proteins are involved in apoptotic processes including the pro-apoptotic protein BAX and the anti-apoptotic proteins Bcl-2 (Table 2). Other targets implicated in apoptosis regulation included the VDAC1 and hexokinase 1 proteins. VDAC1 forms pores in mitochondrial membranes from which apoptotic signaling proteins such as cytochrome c are released [32], whereas binding of hexokinase 1 to VDAC1 affects release of apoptotic proteins and promotes cellular survival [33].

As also suggested by the gene ontology analysis (Figure 3B) the down-regulated proteins typically are involved in cell division, as well as in DNA replication, transcription, repair, and translation (Table 3). Generally, these observations indicate that celastrol-treated cells cut the resources used to general housekeeping functions and proliferation while devoting more resources to cytoprotection and stress handling.

### Validation of MS-based quantification by Western blotting

**Figure 4:**
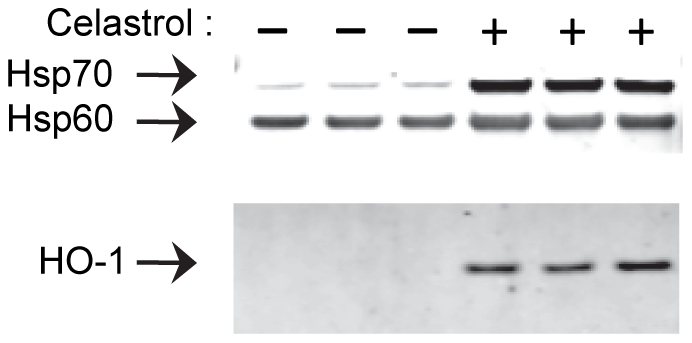
Validation of MS-based protein quantification by Western blot analysis of selected proteins. The soluble protein extracted from cells treated with celastrol or vehicle alone (three cell culturing experiments of each) were separated by SDS-PAGE, blotted onto membranes, and detected using primary antibodies against Hsp70, HO-1, or Hsp60, and fluorescently-labeled secondary antibodies.

To validate the MS-based protein quantification, we performed Western blot analysis of three selected proteins from treated and untreated cells (Figure 4). A visual inspection of the Western blots indicated a good correspondence to data from the MS-based quantification both for the highly celastrol-induced proteins HO-1 (25-fold up-regulated in MS) and Hsp70 (6.7-fold), and for the moderately induced Hsp60 protein (1.6-fold).

### Transcriptional activation of selected genes involved in stress responses and antioxidant defense

**Figure 5:**
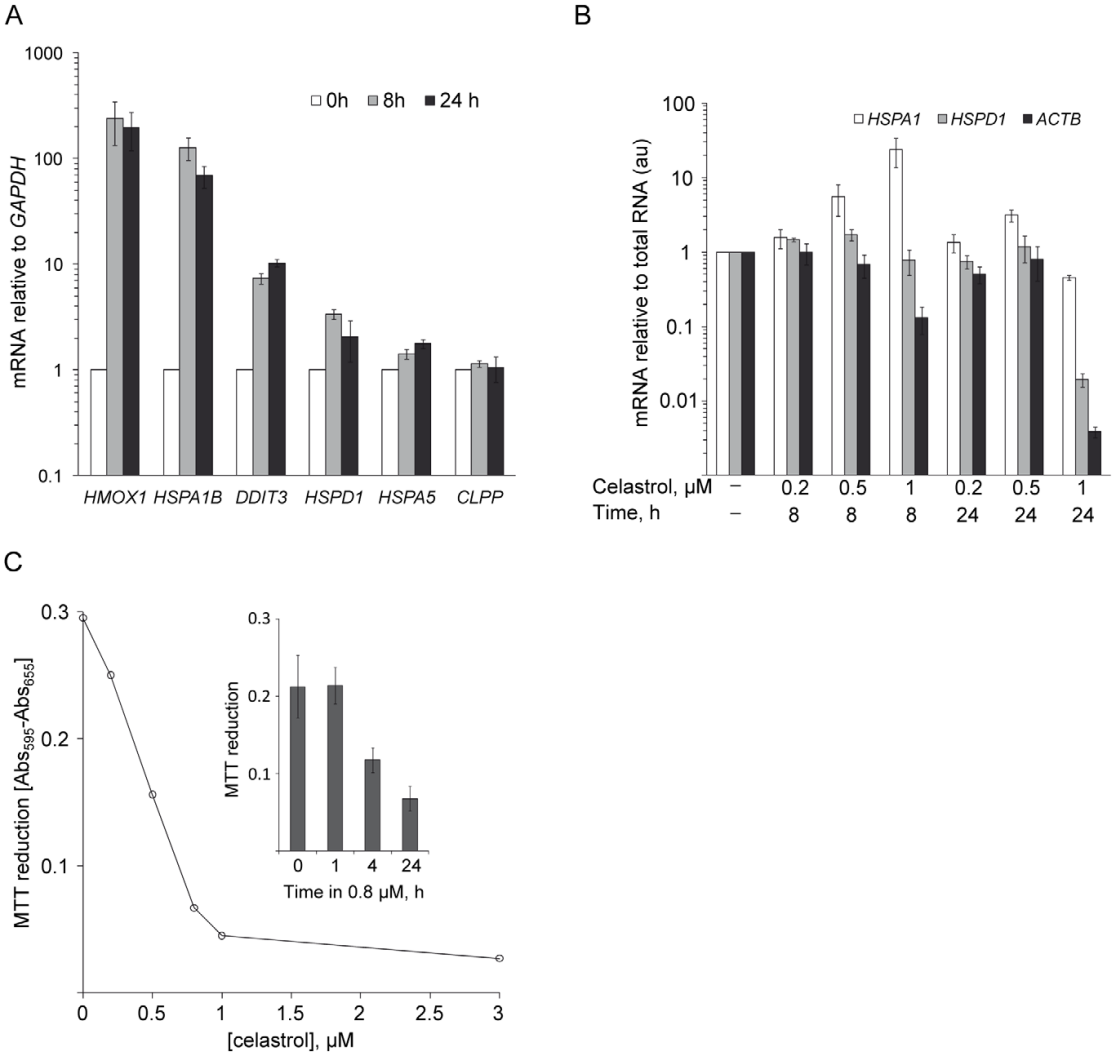
Time- and dose-dependent effects of celastrol on transcriptional activation and cellular viability activity. (A) Transcript levels from genes involved in the heat shock response, ER unfolded protein response, anti-oxidant response, and mitochondrial unfolded protein response. The mRNA levels were measured by quantitative RT-PCR following incubation for 8 or 24 h in 1 µM celastrol. Relative mRNA levels were normalized using *GAPDH* mRNA as a reference and presented relative to levels in untreated control cells. Data are mean of two independent experiments (error bars show the range) and each cDNA was analyzed in triplicate PCR reactions. (B) Time- and dose-dependent inhibition of general gene transcription. The *HSPA1B*, *HSPD1*, and *ACTB* mRNA levels in cells treated with 0.2, 0.5, or 1 µM celastrol for either 8 or 24 h were measured by quantitative RT-PCR. The mRNA levels were normalized to the amount of total RNA used for the RT-PCR reaction and presented relative to levels in untreated cells (arbitrary units, au). Data are mean of two independent experiments (error bars show the range) and each cDNA was analyzed in triplicate PCR reactions. (C) Dose-dependent inhibition of MTT metabolic activity. The metabolic activity/viability following 24 h treatment with varying celastrol concentrations was addressed by quantifying the cellular reduction of MTT into formazan. The amount of MTT reduced in a 4 h period was measured spectrophotometrically by the absorbance Abs [A_595_–A_655_] of the formazan product. Each datapoint are the mean of three experiments. Insert: Metabolic activity of cells exposed to 0.8 µM celastrol for 0, 1, 4, and 24 h. Data are mean (+/−SD) of three experiments.

We investigated whether changes in protein levels were due to transcriptional regulation by analyzing the mRNA levels of selected genes by quantitative reverse-transcriptase PCR (RT-PCR). We focused on genes involved in various cellular stress responses, including *HSPA5* and *DDIT3*/CHOP (ER unfolded protein response), *CLPP* (mitochondrial unfolded protein response), *HMOX1* (antioxidant response), and *HSPA1B*, *HSPD1* (heat shock response). We used 1 µM celastrol to induce a strong transcriptional response (see Figure S1) and measured mRNA levels after 8 and 24 h incubations. We found increased *HSPA1B*, *HSPD1*, *HSPA5*, and *HMOX* mRNA levels (Figure 5A) corresponding to the up-regulated proteins Hsp70, Hsp60, GRP78, and HO-1, respectively (Table 2). This is in accordance with activation of the heat shock response, ER unfolded protein response, and the antioxidant response at the transcriptional level. Celastrol has previously been reported to activate the heat shock response [13] and the ER unfolded protein response [9] in mammalian cells. A study in yeast cells (*Saccharomyces cerevisiae*) has furthermore demonstrated that celastrol triggers the yeast antioxidant responses through activation of the yeast antioxidant transcription factor Yap1 [34].

In contrast, *CLPP* mRNA (Figure 5A) and protein levels (Table S5) were not up-regulated, indicating that celastrol did not activate the mitochondrial unfolded protein response. Celastrol increased the levels of *DDIT3* mRNA encoding the ER stress-activated and apoptosis-regulating transcription factor CHOP [35].

We established that 8 h incubation was sufficient to induce the selected stress genes (Figure 5A) and further studies showed that 4 h incubation was sufficient to significantly increase *HSPA1B* and *HSPD1* mRNA levels (data not shown). The target genes are thus transcribed at a high level several hours prior to the time of proteome analysis.

We noticed that transcript levels across all investigated genes were consistently lower in celastrol-treated cells despite using the same amount of total RNA in RT-PCR reactions. A lower mRNA to total RNA ratio may suggest that celastrol generally inhibits gene transcription. To address this further we re-analyzed the quantitative RT-PCR data by normalizing the mRNA levels from specific genes to the amount of total RNA instead of using a reference gene (*GAPDH*). We found lowered mRNA level from several genes involved in various cellular functions including *ACTB* (encoding the beta-actin protein) (Figure 5B), *ACADM*, and *PGK1* (data not shown) in treated cells. Even the mRNA levels from the highly induced *HSPA1B* and *HSPD1* genes declined at longer exposure times (Figure 5B). This indicates that celastrol, besides inducing the transcription of specific stress response genes, generally inhibits gene transcription in a time- and dose-dependent manner.

The celastrol dose (0.8 µM) used throughout the proteomics studies was selected to give large expression level changes and to facilitate identification of a high number of celastrol-regulated proteins. Using the MTT metabolic activity assay, we found that 0.8 µM celastrol significantly reduced cellular viability. The viability decreased in a concentration-dependent manner reaching a maximum inhibitory effect at 1 µM with 24 h exposure time (Figure 5C). We further established that 4 h exposure to 0.8 µM was sufficient to lower cellular viability (Figure 5C).

### Increased mitochondrial association of selected proteins in celastrol treated cells

**Figure 6:**
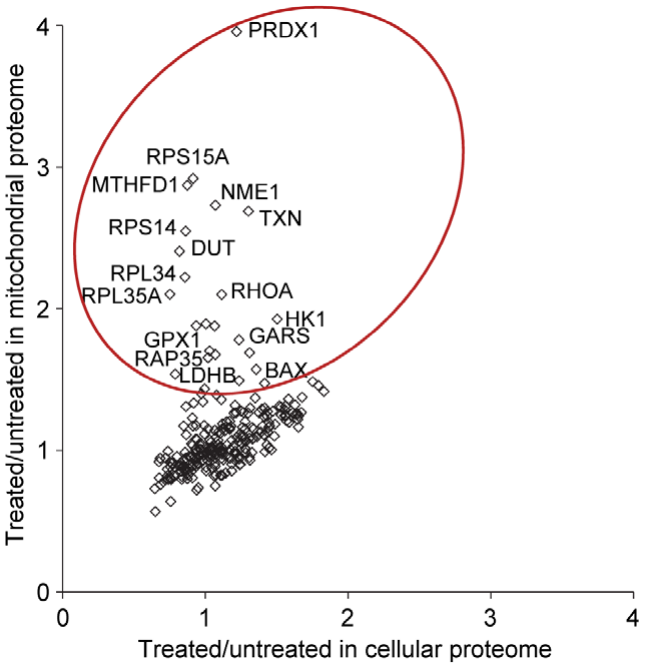
Increased mitochondrial association of selected proteins in response to celastrol. The mean ratio of the 253 core proteins shared by the mitochondrial proteome and the cellular proteome data sets were extracted and presented in a scatter plot. Selected proteins are labeled and putative translocating proteins are enclosed by a circle.

The fact that several proteins were analyzed in both the cellular proteome and the mitochondrial proteome prompted us to investigate if celastrol affected mitochondrial association of a selection of cellular proteins. Theoretically, proteins that associate more with mitochondria in response to celastrol will display a relatively larger treated/untreated ratio when analyzed in isolated mitochondria (mitochondrial proteome experiments) than when analyzed in the cellular proteome experiments. From cross reference of the quantitative data for the 253 core proteins listed in MitoCarta that were quantified in both the mitochondrial and cellular proteome experiments, we identified more than 20 putative mitochondria translocating proteins (Figure 6). These included antioxidant proteins such as peroxiredoxin 1 (*PRDX1*), several ribosomal proteins, hexokinase 1 (*HK1*), BAX, and L-lactate dehydrogenase B (*LDHB*) (Figure 6). Some of the putative translocating proteins identified in this study have previously been reported to associate with mitochondria under various physiological conditions. Peroxiredoxin 1 has classically been categorized as a cytosolic protein, but our data and those of Pagliarini [17] suggest that this protein may associate with mitochondria under specific cellular conditions. Hexokinase 1 can bind to the outer mitochondrial membrane as part of a process that couples the overall rate of glucose metabolism to the rate of mitochondrial oxidative phosphorylation [36], and this association is involved in the regulation of apoptosis and cell survival [33]. The pro-apoptotic protein BAX translocates to mitochondria in response to apoptotic stimuli [37].

### Stress response activation in cell protection and destruction

**Figure 7:**
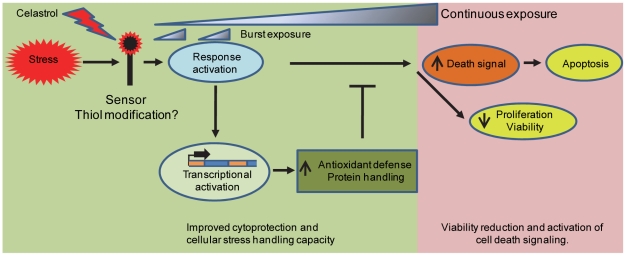
Celastrol effect model. A model illustrating how celastrol-mediated induction of stress response pathways may adapt cells to handle protein misfolding stress and maintain homeostasis or activate cell destructive pathways.

It is clear from this and previous studies [9], [13], [34] that celastrol activates several stress response pathways and antioxidant systems. This may enhance the cellular capacity to handle protein misfolding and damages related to changes in the cellular redox balance as well as offering increased cytoprotection towards acute stress stimuli (Figure 7). However, sustained activation of stress response pathways as e.g. associated with prolonged celastrol exposure may be detrimental to cellular function and cause cell death (Figure 7). Particularly, prolonged activation of the ER unfolded protein response has been associated with apoptotic cell death [24], [38]. It has been suggested that the severity of the ER stress determines whether cells survive or die and that this relies on induction and differential stability of both pro-survival and pro-apoptotic proteins [39]. Up-regulation of stress proteins and of apoptosis-regulating proteins (such as BAX and Bcl-2) by celastrol may thus reflect activation of both adaptive and cell destructive pathways (Figure 7). Supported by the CHOP-activating activity of celastrol observed in this and other studies [9], [34], [40] as well as by the fact that CHOP is believed to play a central role in mediating apoptosis in response to prolonged ER stress [35], we speculate that this transcription factor is fundamental for celastrol-induced cell death. CHOP most likely mediates its effects by activating downstream components such as the ER oxidase 1α protein [41], which we also identified as a celastrol target.

The mechanism by which celastrol activates multiple cellular pathways is still uncertain, but its ability to react with thiol groups of regulatory proteins possibly plays a key role [34], [42]. If the thiol-reactivity of celastrol is more prevalent, one may speculate that it interferes with disulfide bridge formation and correct folding of non-native proteins. In the ER, where the flow of non-native proteins is high, this may lead to accumulation of misfolded proteins and activation of the ER unfolded protein response. Whether this mechanism can explain the significant impact of celastrol on the ER quality control system observed in this study is unclear, but could be the subject of future studies.

Interestingly, a molecular mechanism responsible for the activity of triptolide, another active compound derived from *Tripterygium wilfordii* with potential anti-cancer activities, has recently been reported. Triptolide modifies the activity of the transcription factor TFII through specific and covalent binding to the XPB subunit [43]. One consequence of triptolide-XPB binding is general inhibition of the RNA polymerase II mediated transcription, which resembles the general reduction of the level of several mRNA observed in the celastrol-treated cells of this study. Triptolide and celastrol both have anti-proliferative and apoptosis-inducing activities, and are therefore recognized as prime candidates for the development of anti-cancer drugs from traditional Chinese herbal medicine [7], [44]. However, triptolide and celastrol differ in the way heat shock gene expression is affected. Triptolide inhibits the heat shock response by impairing the transactivation function of the transcription factor hsf1 [45], whereas celastrol activates the heat shock response through activation of hsf1 [34]. Specific targets of celastrol that may explain its anti-proliferative and apoptosis-inducing activities have now been revealed in this (Figure 7) and other recent investigations, see e.g. [10], [40], [46]–[48]. This may facilitate complete unraveling of the molecular mechanism of celastrol but also pinpoint relevant anti-cancer pathways that may be targeted more specifically by other drugs. However, it is crucial that these targets and their significance are validated in relevant *in vivo* cancer models before initiation of clinical trails. These investigations should include careful evaluation of pharmacokinetic parameters as we have observed cell specific differences in celastrol dose response with regard to both the toxicity and stress response induction (compare Figures 5 and S3). Therapeutic usage of celastrol for cytoprotection and improvement of cellular protein homeostasis requires that severe anti-proliferative and cell-death activating effects can be avoided through implementation of appropriate treatment regime. One way to address this could be to investigate whether short bursts of celastrol exposure may increase the protein handling capacity sufficiently to enhance cellular clearance of misfolded proteins while simultaneously avoiding unwanted side effects observed during prolonged exposure (Figure 7).

In conclusion, we have provided an extensive biomarker catalog of celastrol-regulated proteins and revealed multifaceted cellular effects that can facilitate development and monitoration of treatment regimes based on celastrol or a more selective chemical derivative.

## Materials and Methods

Sections of material and methods are described more detailed in Text S1.

### Cell culture and SILAC labeling

Lymphoblastoid cells (Coriell Institute for Medical Research USA) were grown at 37°C in 95% humidified air and 5% (v/v) CO2 in culture flasks (TPP). Culture media for SILAC labeling were prepared according to guidelines in [49] using arginine and lysine-depleted RPMI media (Pierce) supplemented with 10% dialyzed fetal calf serum (Invitrogen), penicillin, streptomycin (both Leo Pharmaceutical Products), and either 35 mg/l standard arginine and 50 mg/l standard lysine (Invitrogen) or molar equivalents of heavy isotopic arginine (Arg10: [^13^C_6_, ^15^N_4_]-L-Arginine) and lysine (Lys6: [^13^C_6_] L-Lysine). Celastrol (Gaia Chemical Corporation, USA) was added to a final concentration of 0.8 µM to cells grown in heavy isotopic media for 6 cell-doublings and equivalent amount of vehicle alone (dimethyl sulfoxide) was added to cells grown in standard isotopic media. Twenty-four hours later, celastrol-treated and untreated cells were collected and proteins were extracted according to two different protocols for analysis of either the cellular proteome or the mitochondrial sub-proteome (see below and Figure 1).

### Sample preparation for MS analysis

For analysis of the cellular proteome, equal amounts of protein (see Text S1 for details on protein extraction and cell lysis) from celastrol- and DMSO-incubated cells (20 µg from each) were mixed at a 1∶1 ratio and separated on a 12% sodium dodecyl sulfate-bis-Tris polyacrylamide gel (SDS-PAGE) (Criterion XT; BioRad). For analysis of the mitochondrial proteome, a mitochondria-enriched protein fraction was isolated (Qproteome mitochondrial isolation kit, QIAGEN) from a 1∶1 mixture of treated and untreated cell populations, and a 30-µg aliquot was separated by SDS-PAGE. Each lane in the gel was divided into 12 (mitochondrial proteome) or 16 (cellular proteome) samples. Each sample was reduced for 10 min at 60°C in freshly prepared 50 mM tris(2-carboxyethyl)phosphine (TCEP), and then alkylated in 100 mM iodoacetamide for 50 min in the dark. Following two 15-min wash steps at 37°C in 50% acetonitrile (AcN), each protein sample was digested with 0.6 µg trypsin (Trypsin Gold; Promega) overnight at 30°C in 2.25 mM ammonium bicarbonate (pH 8.2). Peptides were extracted from the gel into protein low-binding tubes (Eppendorf) in two consecutive steps, first with 80% AcN and 1% trifluoracetic acid (TFA), and then with 100% AcN. Peptides were purified using PepClean C-18 Spin Columns (Pierce) according to the manufacturer's protocol, lyophilized, and then re-dissolved in 40 µl MS buffer A containing 0.4% acetic acid and 5% AcN before analysis by nanoLC-MS/MS.

### NanoLC-MS/MS analysis

The peptide mixtures were analyzed by liquid chromatography (Easy nLC; Proxeon) coupled to mass spectrometry (LTQ-Orbitrap; Thermo Fisher Scientific) through a nano-electrospray source (Proxeon) essentially as described previously [50]. The reverse phase separation column, 10 cm long and 75 µm in inner diameter (G&T Septech), was packed with 3.5 µm Kromasil C18 particles (Eka Chemicals). The peptides were separated in a 100-min gradient of AcN in 0.4% acetic acid, starting with 5% and ending with 36% AcN. MS detection was full scan (m/z 400–2000) with Orbitrap detection at resolution R = 60,000 (at m/z 400) followed by up to four data-dependent MS/MS scans with LTQ detection of the collision-induced dissociation (CID) fragments of the most intense parent ions. Dynamic exclusion of 25 s was used as well as rejection of a charge state of +1.

### Database searches and statistics

Mascot version 2.2.04 (Matrix Science) was used for peptide identification and MaxQuant version 1.0.13.13 [16] for protein identification and quantification. The MS data were searched against IPI protein database version 3.52 containing 73,928 sequences and the same number of reversed sequences for false discovery rate calculations (FDR). FDR was set to 0.01 for both identification of peptides and proteins, corresponding well with the identification of 36 proteins in the reversed database out of 3660 identified proteins in total. MS/MS mass tolerance was 0.5 Da. Setting of trypsin digestion was cleavage at C-terminus of lysine and arginine except before proline, and up to two missed cleavages were accepted. Carbamidomethylation at cysteine residues was set as fixed modification and oxidation of methionine was set as variable modification. Only peptides with a minimum length of 6 amino acids were accepted and at least two peptides (and one unique peptide) were required for protein identification. Calculations of quantitative SILAC protein ratios (treated/untreated) were performed with Maxquant algorithms using standard settings [16]. The resulting identification and quantification data are listed in Tables S7 (protein data) and S8 (peptide data). A median SILAC protein ratio was calculated from at least two valid peptide ratio counts. Normalized protein ratios were calculated in MaxQuant software using peptide ratios normalized within each MS-run by setting the median of their logarithm to a value of zero.

### Gene ontology (GO) analysis

The distribution of the up- and down-regulated proteins into GO categories was compared pairwise to the distribution of all quantified members of the cellular proteome study analyzed by Pearson Chi-Square statistics using the web-based WeGo tool [51]. The analysis was restricted to the GO categories hosted one and two levels below the general GO category “biological process”. The up- and down-regulated proteins were defined as the cellular core proteins for which the mean treated/untreated ratio was different from 1 (Student's t-test at p<0.05).

### Quantitative RNA analysis and Western blotting

RNA isolated from cells using the Total RNA isolation system (Promega) was analyzed by quantitative reverse-transcriptase PCR. RNA was transcribed into cDNA (Advantage RT for-PCR Kit, Clontech) and analyzed by real-time PCR using Taqman probe chemistry (Applied Biosystems). Relative quantification with GAPDH as the reference gene was performed as previously described [52] using the standard curve method with a calibration curve constructed from a 10–fold dilution series of pooled cDNA.

For Western blotting, soluble proteins extracted from cells by a detergent-based lysis buffer and centrifugation were transferred to a polyvinylidene fluoride membrane (Millipore) using a semidry blotting system (Bio-Rad). Proteins were detected by primary antibodies against the proteins Hsp60 (H-3524; Sigma), HO-1 (OSA-110; stressgen), and Hsp70 (SPA810; Stressgen) using infrared dye-labeled secondary antibodies (IRDye 800CW and IRDye 680, LI-COR Biosciences) and an infrared imaging system (Odyssey; LI-COR Biosciences).

### Cellular viability assay

Cellular viability was addressed by the cellular reduction of MTT (3-(4,5-dimethylthiazol-2-yl)-2,5-diphenyltetrazolium bromide) assay [53] as described previously [54].

## Supporting Information

Figure S1 **Celastrol dose response in lymphoblastoid cells evaluated by** ***HSPA1B*** **and** ***HSPD1*** **mRNA levels.** The dose-dependent induction of *HSPA1B* and *HSPD1* mRNA (encoding the Hsp70 and Hsp60 proteins, respectively) were analyzed by quantitative RT-PCR using RNA isolated from lymphoblastoid cells treated with varying celastrol concentrations for 24 h. The relative mRNA levels from different genes were normalized using *GAPDH* mRNA as reference and presented relative to untreated control cells (t = 0). Data are mean of two independent experiments (errors bars show the range) and each cDNA was analyzed in triplicate PCR reactions.
(TIF)

Figure S2 **Variation between replicates in the cellular proteome study.** Coefficient of variation (values in 10% intervals) for the quantitative ratios (treated/untreated) for the 1779 cellular core proteins illustrated by frequency histogram.
(TIF)

Figure S3 **Cell specific celastrol toxicity and induction of heat shock gene expression.** (A) Comparison of the dose dependent toxicity of celastrol in HEK293 and lymphoblastoid cells measured by the MTT assay. Viability of cells incubated for 24 h in celastrol expressed relative to cells incubated in vehicle alone (DMSO). (B) The inducible expression of markers of the heat shock response (*HSPA1B*) and ER UPR (*DDIT3*) in HEK293 following 24 h incubation in celastrol analyzed by quantitative RT-PCR as described in figure 5A legend.
(TIF)

Table S1 **All quantified cellular proteins.** This table lists the treated/untreated quantitative ratios for all 2765 different proteins quantified in at least one of three replicated cellular proteome experiments.
(XLS)

Table S2 **Cellular core proteins.** This table shows selected data (including the quantitative ratio (treated/untreated) for single experiments, mean ratio, and descriptive statistic) for the 1779 cellular core proteins quantified repeatedly in three cellular proteome experiments.
(XLS)

Table S3 **Cellular core proteins 1.5 fold.** This table lists the 158 cellular core proteins that are altered at least 1.5 fold.
(XLS)

Table S4 **All mitochondrial proteins.** This table lists the treated/untreated quantitative ratio for the 498 quantified proteins that are classified as mitochondrial by their listing in Human MitoCarta.
(XLS)

Table S5 **Mitochondrial core proteins.** This table shows selected data (including the quantitative ratio (treated/untreated) for single experiments, mean ratio, and descriptive statistic) for the 374 mitochondrial core proteins quantified repeatedly in three mitochondrial proteome experiments.
(XLS)

Table S6 **Mito core proteins 1.5 fold.** This table lists the 33 mitochondrial core proteins that are altered at least 1.5 fold.
(XLS)

Table S7 **Protein evidence files.** The table shows output from Maxquant's “Protein Groups” and contains data for all six experiments, three cellular proteome experiments (D, E, and F), and three mitochondrial proteome experiments (A, B, and C). Numbers in the “Peptide ID” column refers to table S8.
(XLS)

Table S8 **Peptide evidence files.** This table shows peptide evidence output from MaxQuant for identified proteins in table S7.
(XLS)

Text S1 **Supplementary materials and methods.** Detailed description of selected materials and methods.
(DOC)
