# Supplementary figures and images for: Quantitative Proteomics Reveals Cellular Targets of Celastrol
Source: PLoS One. 2011 Oct 26;6(10):e26634. doi: 10.1371/journal.pone.0026634 (PMC3202559; doi:10.1371/journal.pone.0026634)

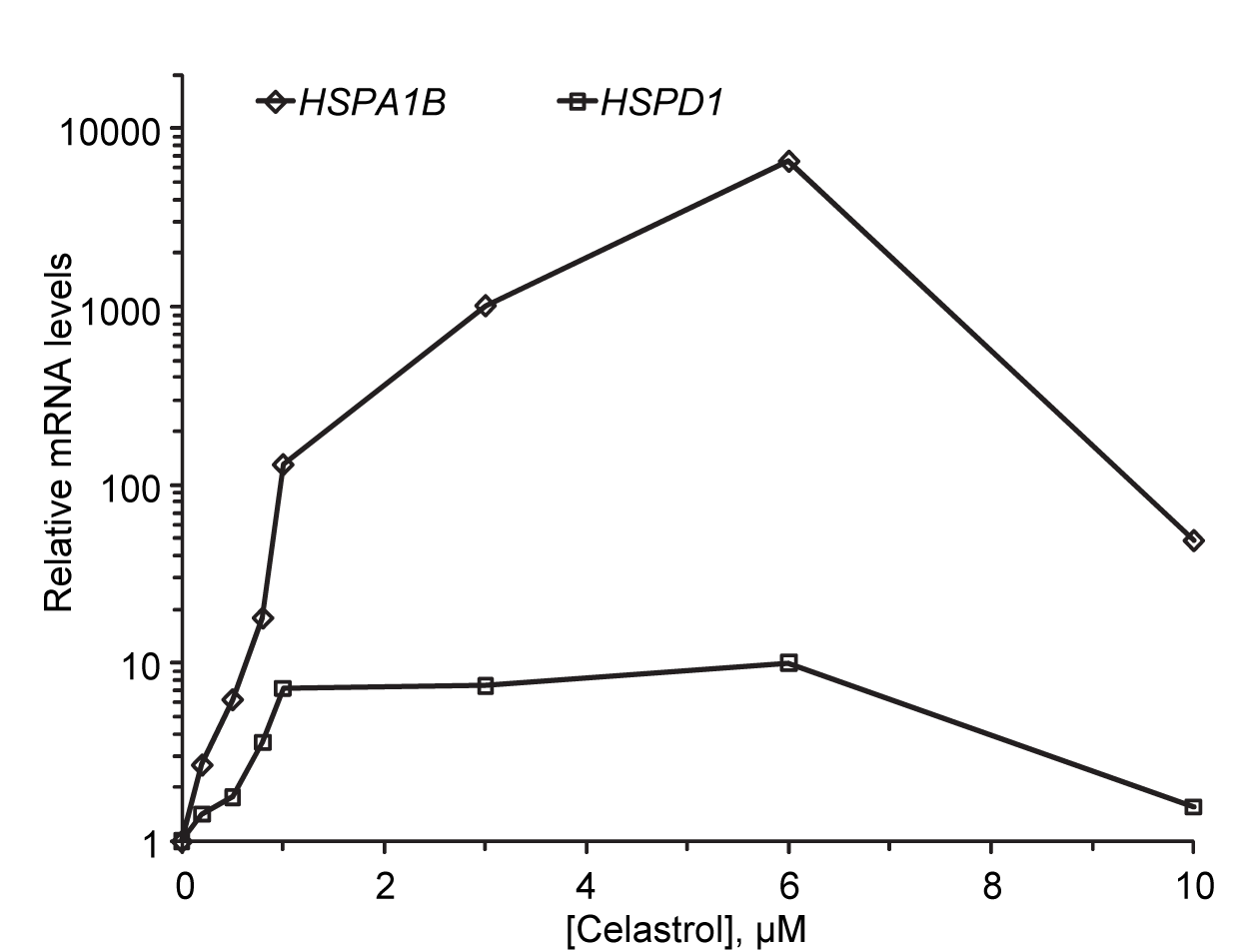

Supplement: Figure S1 — Celastrol dose response in lymphoblastoid cells evaluated by HSPA1B and HSPD1 mRNA levels. The dose-dependent induction of HSPA1B and HSPD1 mRNA (encoding the Hsp70 and Hsp60 proteins, respectively) were analyzed by quantitative RT-PCR using RNA isolated from lymphoblastoid cells treated with varying celastrol concentrations for 24 h. The relative mRNA levels from different genes were normalized using GAPDH mRNA as reference and presented relative to untreated control cells (t = 0). Data are mean of two independent experiments (errors bars show the range) and each cDNA was analyzed in triplicate PCR reactions. (TIF) [file pone.0026634.s001.tif]

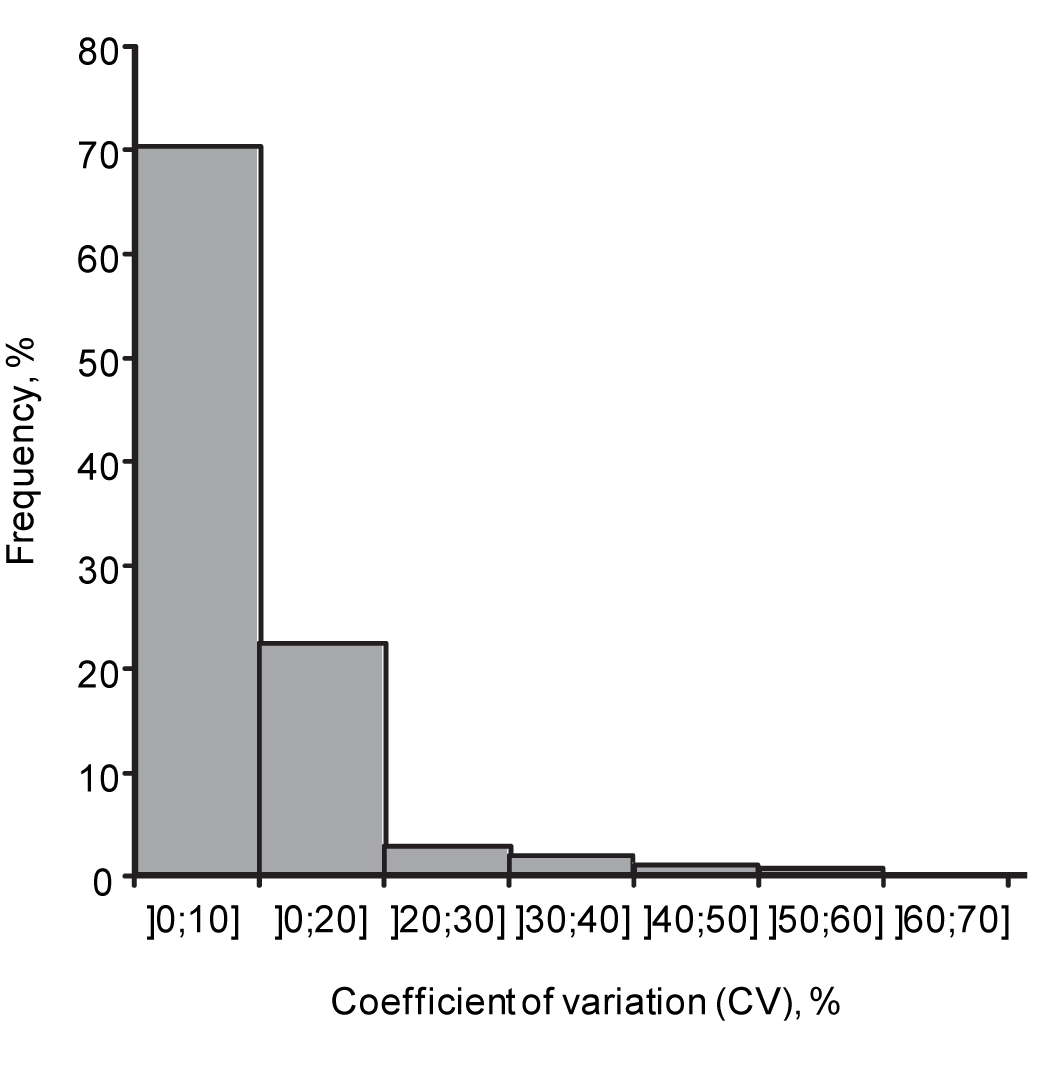

Supplement: Figure S2 — Variation between replicates in the cellular proteome study. Coefficient of variation (values in 10% intervals) for the quantitative ratios (treated/untreated) for the 1779 cellular core proteins illustrated by frequency histogram. (TIF) [file pone.0026634.s002.tif]

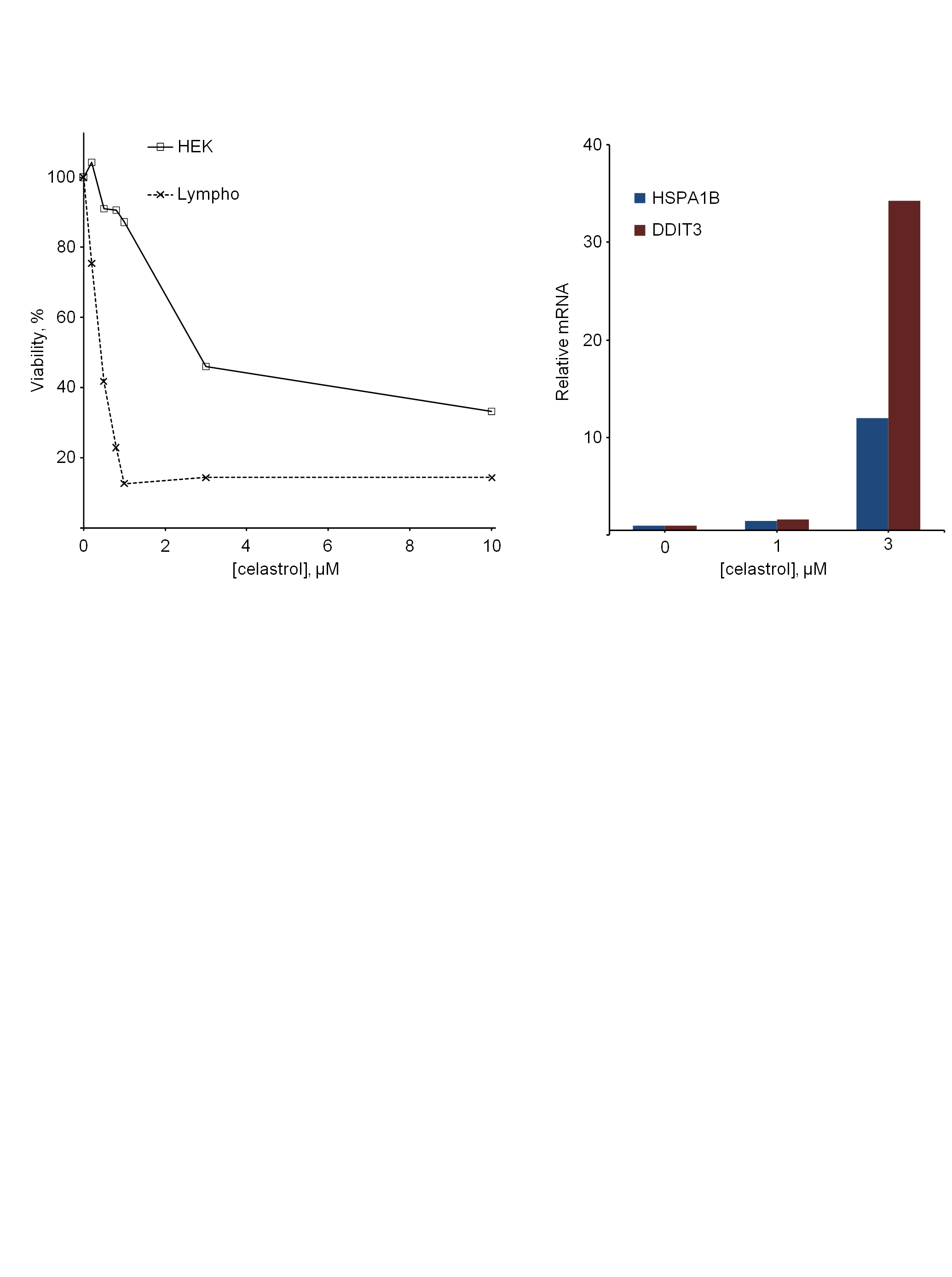

Supplement: Figure S3 — Cell specific celastrol toxicity and induction of heat shock gene expression. (A) Comparison of the dose dependent toxicity of celastrol in HEK293 and lymphoblastoid cells measured by the MTT assay. Viability of cells incubated for 24 h in celastrol expressed relative to cells incubated in vehicle alone (DMSO). (B) The inducible expression of markers of the heat shock response (HSPA1B) and ER UPR (DDIT3) in HEK293 following 24 h incubation in celastrol analyzed by quantitative RT-PCR as described in figure 5A legend. (TIF) [file pone.0026634.s003.tif]
