# Supplementary material for: Quantitative Proteomics Reveals Cellular Targets of Celastrol
Source: PLoS One. 2011 Oct 26;6(10):e26634. doi: 10.1371/journal.pone.0026634 (PMC3202559; doi:10.1371/journal.pone.0026634)
Supplement: Text S1 — Supplementary materials and methods. Detailed description of selected materials and methods. (DOC) [file pone.0026634.s012.doc]

**Text S1. Supplementary materials and methods**

**Reagents**

All standard chemicals were obtained from Sigma/Fluka (Sigma-Aldrich) unless otherwise specified. Celastrol was obtained from Gaia Chemical Corporation, dissolved in dimethyl sulfoxide (DMSO), and stored as 10-mM aliquots at -20°C.

**Cell culture**

The lymphoblastoid cell line used throughout the study was obtained from the “apparently healthy collection” at Coriell Institute for Medical Research USA. Cells cultured for all non-SILAC experiments were grown in RPMI-1640 medium (BioWhittaker) supplemented with 10% fetal calf serum (Biological Industries), 100 U/ml penicillin and 0.1 mg/ml streptomycin (both Leo Pharmaceutical Products). Cells used for RNA and Western blotting analysis were seeded at a density of 2×10^5^ cells per ml in culture flasks (TPP) positioned upright, and then were harvested 2 days later by centrifugation, washed twice in phosphate buffered saline (PBS), and frozen immediately at -80°C.

**SILAC labeling**

We used a standard isotopic arginine concentration of 35 mg/L (and equivalent molar amounts of heavy isotopic arginine) to minimize arginine to proline conversions and a lysine concentration of 50 mg/L. These concentrations of arginine and lysine were tested and confirmed not to inhibit cellular growth and resulted in negligible amounts of arginine to proline conversions. The protein-labeling efficiency was estimated by analyzing MS data (using MaxQuant software as described in material and methods section of the main text) of proteins extracted from cells grown in heavy isotopic media for 6-cell doublings. The intensity of the heavy isotopic peptides constituted >95% of the total peptide intensities in all of three extracted protein samples from different cell cultures.

Cells for SILAC labeling experiments were seeded at an initial density of 1.5×10^5^ cells/ml in media containing either standard or heavy isotopic variants of arginine and lysine in T25 culture flasks (TPP) and allowed to proliferate to a density of 6×10^5^ cells/ml. Following gentle collection of cells by centrifugation and removal of old medium, the cells were subsequently re-dissolved in the appropriate type of fresh medium to the initial density of 1.5×10^5^ cells/ml and then transferred to T150 culture flasks for further proliferation. This re-feeding and cell dilution cycle was repeated until each of the cell cultures had undergone at least six doublings as verified by cell counts using an automated cell counter (Nucleocounter, Chemometec). Celastrol was then added to a final concentration of 0.8 µM to cells grown in heavy isotopic media and equivalent amounts of vehicle only (DMSO) was added to cells grown in standard isotopic media (final amount of DMSO less than 0.08%). Twenty-four hours later, celastrol- and vehicle-incubated cells were collected, and then subjected to either a total cell proteome approach or a mitochondrial-focused approach (mito-focus). For the total proteome approach, celastrol- and vehicle-incubated cells were harvested separately by centrifugation, washed twice in PBS buffer, and lysed by three freeze/thaw cycles in a buffer containing 50 mM Tris-HCl (pH 7.7), 5 mM EDTA, 1% Triton X-100, 1 mM DTT, and protease inhibitor (complete mini tablet, Roche Applied Science) followed by a 30-s burst in a cooled water bath sonicator (Branson, Ultrasonic cleaner). The resulting cell extracts were centrifuged for 20 min at 15,000×*g*, pellets were discarded, and the collected supernatants were snap frozen at -80°C after the protein concentration had been determined by the Bradford protein assay (Bio-Rad).

For mito-focus analysis, celastrol- and vehicle-incubated cells were mixed at a 1:1 ratio (4×10^7^ cells of each cell type). A mitochondria-enriched fraction of the cell mixture was prepared using the Qproteome mitochondrial isolation kit according to manufacturer’s instructions (QIAGEN).

**Quantitative RNA analysis**

RNA was isolated from frozen cell pellets using the SV Total RNA isolation system (Promega), which included an on-column DNA digest by deoxyribonuclease I to minimize contaminating DNA. RNA, with an optical density ratio (A_260 nm_/A_280 nm_) above 1.8, was reverse transcribed into cDNA in a 20-µl reaction with 1 µg total RNA and 100 pmol “anchored” oligo(dT) primers (18T+N) using the Advantage RT for-PCR Kit (Clontech) according to the manufacturer’s instructions. Semi-quantitative reverse transcriptase- polymerase chain reaction (RT-PCR) was performed using the ABI7000 real-time sequence detection system and TaqMan probe chemistry (Applied Biosystems). Primers and probes were designed using the primer express software (Applied Biosystems) (sequences available upon request). Aside from the pre-designed assay for *HSPA1B* (Hs00271244_s1, Human HspA1B; Applied Biosystems) all other probes were designed to anneal within an exon-exon junction in order to avoid detection of possible contaminating genomic DNA. PCR and relative quantification using the standard curve method was performed as previously described [1]. All PCR assays used had an amplification efficiency above 90%, as determined from the slope (PCR efficiency= 10 ^(-1/slope)^−1) of the calibration curve used for quantification, and was prepared from a standardized dilution series of a pooled cDNA standard.

**Western blotting analysis**

Protein aliquots (10 µg) were separated on 4-12 % SDS-PAGE gels (Criterion XT, BioRad) and blotted onto polyvinylidene fluoride membranes (Millipore) using a semidry blotting system (Bio-Rad). Membranes were blocked in 5% skim milk in PBS + 0.1% Tween-20, washed in PBS + 0.1% Tween-20, and incubated overnight with primary antibodies against the proteins Hsp60 (H-3524; Sigma), HO-1 (OSA-110; Stressgen), and Hsp70 (SPA810; Stressgen). Bound antibodies were detected using direct infrared fluorescence using infrared dye-labeled secondary antibodies (IRDye 800CW Goat anti-Rabbit IgG and IRDye 680 Goat anti-Mouse IgG; LI-COR Biosciences) and an infrared imaging system (Odyssey; LI-COR Biosciences).

**Cellular viability (MTT reduction assay).**

Cellular metabolic activity (viability) was measured using the cellular MTT (3-(4,5-dimethylthiazol-2-yl)-2,5-diphenyltetrazolium bromide) reduction assay [2] as described previously [3]. Metabolically active cells reduce MTT to an insoluble formazan product that, following solubilization, can be quantified in a microplate reader. Cells were seeded at a density of 1×10^4^ cells per well in 100 µl RPMI-1640 media (BioWhittaker) in a 96-well tissue culture plate (Nunc) and incubated at 37°C in 95% humidified air and 5% (v/v) CO_2_. After 24 h, 10 µl of MTT dissolved in PBS to 5 mg/ml (Roche Applied Science) was added to each well, followed by 100 µl stop/solubilization solution (10% SDS in 0.01 M HCl) 4 h later. The amount of formazan formed was quantified using a microplate reader (iMark, Bio-Rad) measuring the absorbance at 595 nm with reference at 655 nm.

Reference List

1. Hansen J, Corydon TJ, Palmfeldt J, Durr A, Fontaine B, Nielsen MN, Christensen JH, Gregersen N, Bross P (2008) Decreased expression of the mitochondrial matrix proteases Lon and ClpP in cells from a patient with hereditary spastic paraplegia (SPG13). Neuroscience 153: 474-482.

2. Mosmann T (1983) Rapid colorimetric assay for cellular growth and survival: application to proliferation and cytotoxicity assays. J Immunol Methods 65: 55-63.

3. Hansen J, Bross P (2010) A cellular viability assay to monitor drug toxicity. Methods Mol Biol 648: 303-311. 10.1007/978-1-60761-756-3_21 [doi].
